# Supplementary material for: Automatic and unbiased segmentation and quantification of myofibers in skeletal muscle
Source: Sci Rep. 2021 Jun 3;11:11793. doi: 10.1038/s41598-021-91191-6 (PMC8175575; doi:10.1038/s41598-021-91191-6)
Supplement: Supplementary file 1 — Supplementary Information. [file 41598_2021_91191_MOESM1_ESM.pdf]

Supplementary Material for Automatic and unbiased segmentation and quantification of myofibers in skeletal muscle

Ariel Waisman[1,*], Alessandra Norris[2,3], Martín Elías Costa[4] and Daniel Kopinke[2,3,*]

[1]CONICET - Fundación para la Lucha contra las Enfermedades Neurológicas de la Infancia (FLENI), Laboratorio de Investigación Aplicada a Neurociencias (LIAN), Buenos Aires, Argentina. [2]Department of Pharmacology and Therapeutics, University of Florida College of Medicine, Gainesville, FL 32610, USA. [3]Myology Institute, University of Florida College of Medicine, Gainesville, FL, USA. [4]Universidad de Buenos Aires, Buenos Aires, Argentina. [*]Correspondence: awaisman@fleni.org.ar, dkopinke@ufl.edu

1 Supplementary Figures

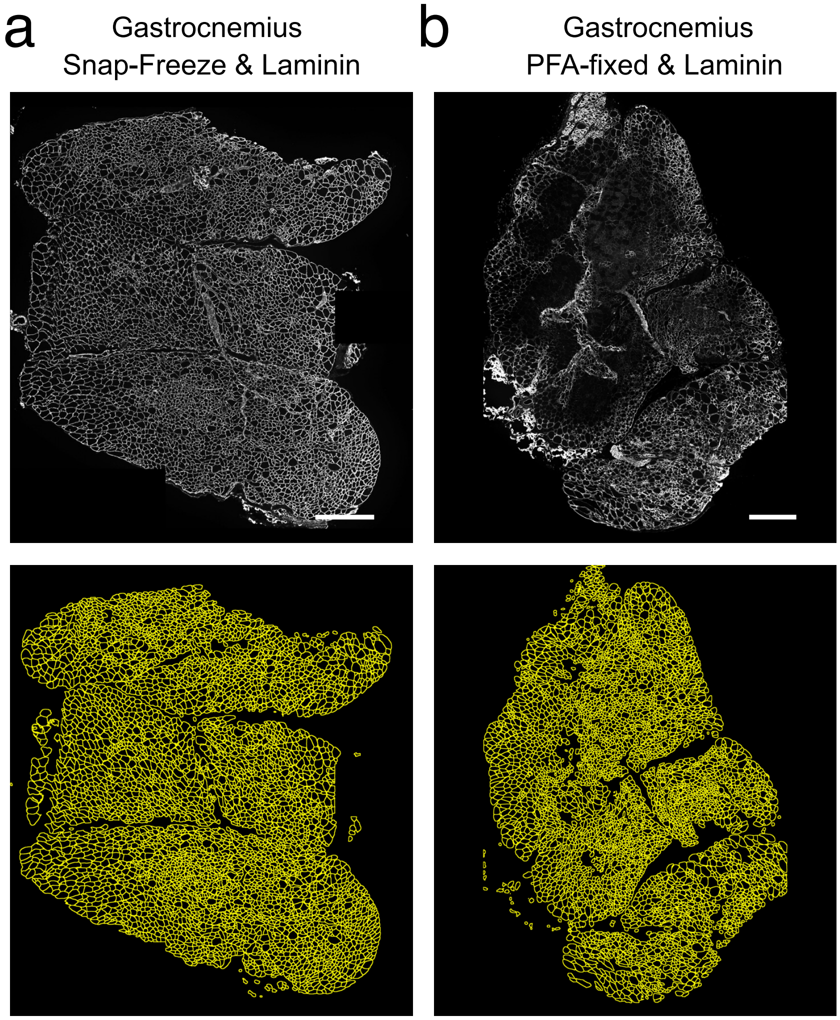

Figure S1: **Cellpose segmentation of gastrocnemius muscle**. Cross section of (a) snap-frozen or (b) PFA-fixed gastrocnemius muscle stained for Laminin. Top, original image. Bottom, Cellpose segmentation results. Scale bar, 500 µm.

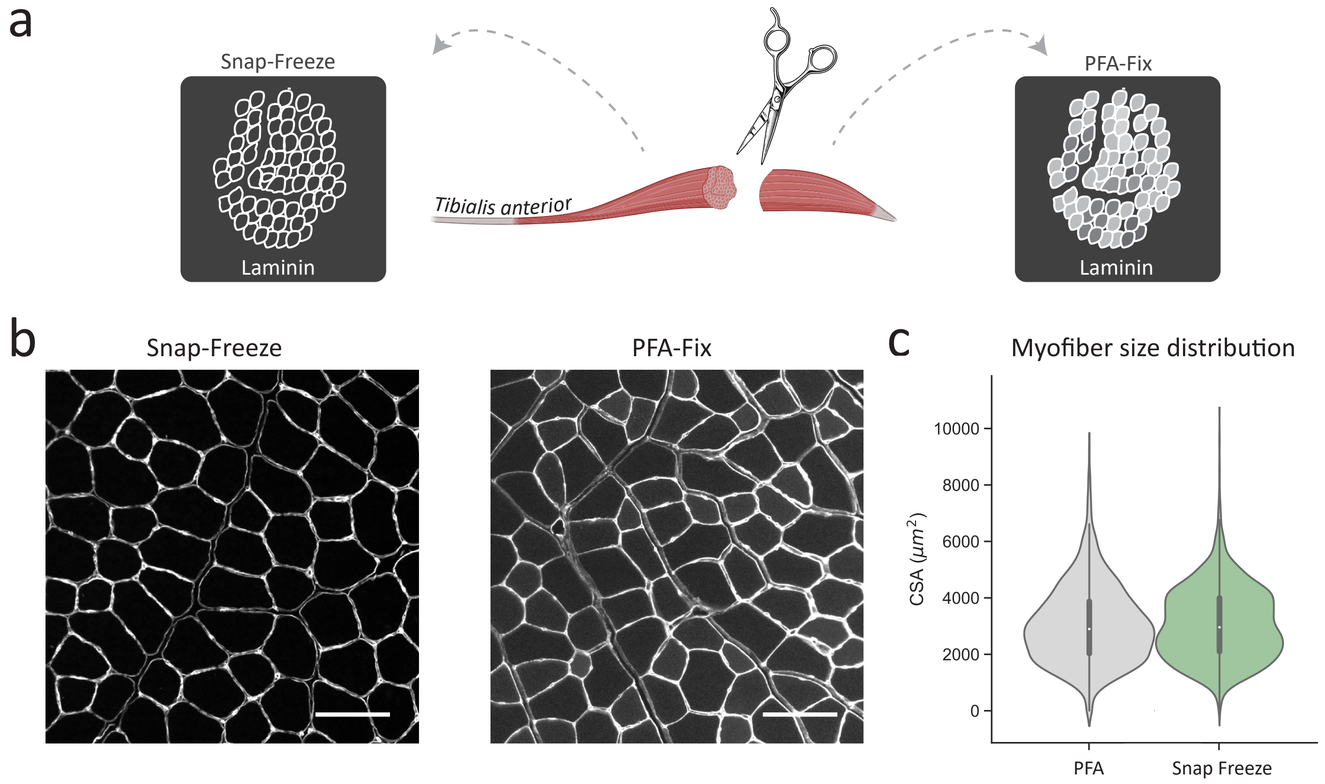

Figure S2: **Fixation does not cause changes in myofiber size.** (a) TA muscles were cut in half with one half immediately snap frozen while the other half was PFA-fixed. (b) Cryosectioned of both pieces of the same TA were stained for Laminin and then run through Cellpose. Scale bar, 100 μm. (c) The mean myofiber size distribution is the same between fixed and snap-frozen muscle tissue.

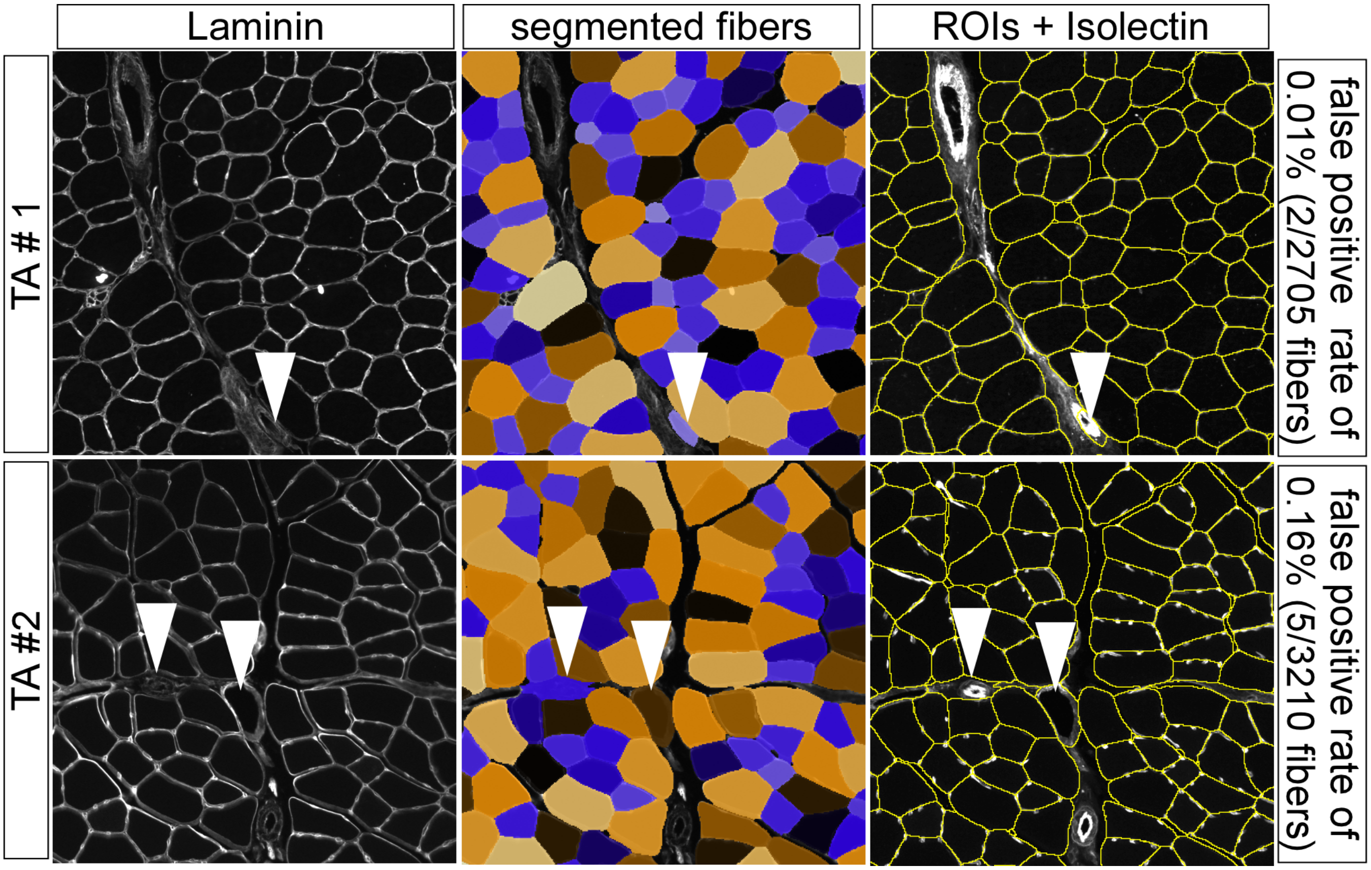

Figure S3: **Cellpose displays a very low false-positivity rate in detecting blood vessels.** Snap-frozen cryosections of two TAs were stained for Isolectin GS-IB4 to mark blood vessels and Laminin to outline the myofibers. After Cellpose segmentation of the Laminin channel, represented as false color coded segmented fibers, the labeled regions of interests (ROIs) were overlayed with Isolectin GS-IB4. Arrowheads highlight blood vessels that were wrongly segmented as myofibers. We detected a total of 2 mis-segmented fibers in one TA and 5 in the other out of 2705 (false-positivity rate of 0.08%) and 3210 total myofibers (false-positivity rate of 0.16%), respectively.

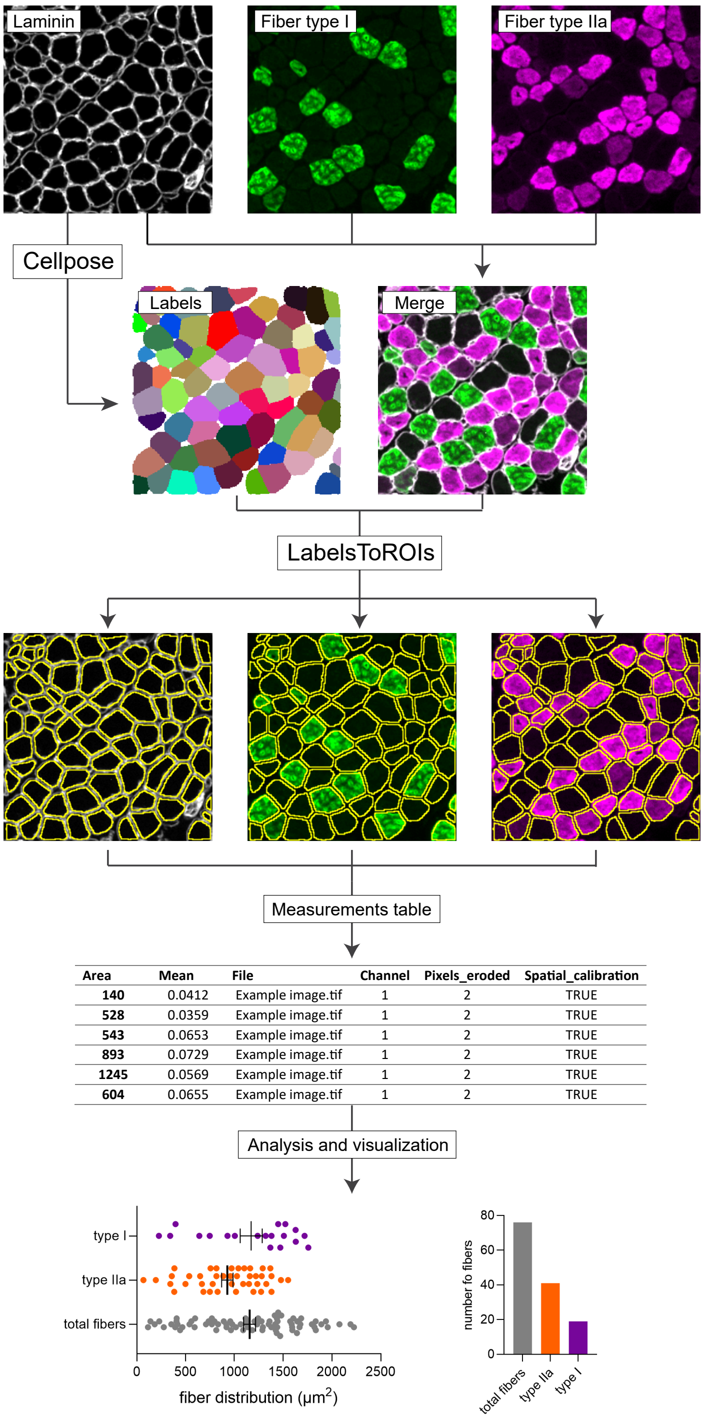

Area	Mean	File	Channel	Pixels_eroded	Spatial_calibration
**140**	0.0412	Example image.tif	1	2	TRUE
**528**	0.0359	Example image.tif	1	2	TRUE
**543**	0.0653	Example image.tif	1	2	TRUE
**893**	0.0729	Example image.tif	1	2	TRUE
**1245**	0.0569	Example image.tif	1	2	TRUE
**604**	0.0655	Example image.tif	1	2	TRUE

Figure S4: **Segmentation pipeline enables simple and automated myofiber type analysis**. Snap frozen gastrocnemius muscle section were stained with Laminin (gray scale) to delineate all fibers, BA-D5 to mark Type 1 fibers (green) and SC-71 for Type IIA fibers (purple). The Laminin image was fed to Cellpose for myofiber segmentation. The resulting labeled image, together with the merged image containing all the channels were fed to the LabelsToROIs plugin for ROI erosion and fluorescence intensity quantification. Analysis of the resulting table in Excel allowed the calculation of myofiber numbers and size distribution.
